# Evolutionary exploration of polytypism in lead halide perovskites†

**DOI:** 10.1039/d1sc03098a

**Published:** 2021-08-10

**Authors:** Zhenzhu Li, Ji-Sang Park, Aron Walsh

**Affiliations:** Department of Materials, Imperial College London London SW7 2AZ UK a.walsh@imperial.ac.uk; Department of Physics, Kyungpook National University Daegu 41566 Korea; Department of Materials Science and Engineering, Yonsei University Seoul 03722 Korea

## Abstract

The regular ABX_3_ cubic perovskite structure is composed of close-packed AX_3_ layers stacked along the 〈111〉 axis. An equivalent hexagonal close-packed network can also be formed, in addition to a series of intermediate polytype sequences. Internally, these correspond to combinations of face- and corner-sharing octahedral chains that can dramatically alter the physical properties of the material. Here, we assess the thermodynamics of polytypism in CsPbI_3_ and CsPbBr_3_. The total energies obtained from density functional theory are used to paramaterize an axial Ising-type model Hamiltonian that includes linear and cubic correlation terms of the pseudo-spin. A genetic algorithm is built to explore the polytype phase space that grows exponentially with the number of layers. The ground-state structures of CsPbX_3_ polytypes are analysed to identify features of polytypism such as the distinct arrangements of layers and symmetry forbidden sequences. A number of polytypes with low ordering energies (around thermal energy at room temperature) are predicted, which could form distinct phases or appear as stacking faults within perovskite grains.

## Abstract 2

Beyond the regular perovskite structure based on cubic-close packing exists a range of possible polytypes that we explore using computational chemistry.

## Graphical abstract

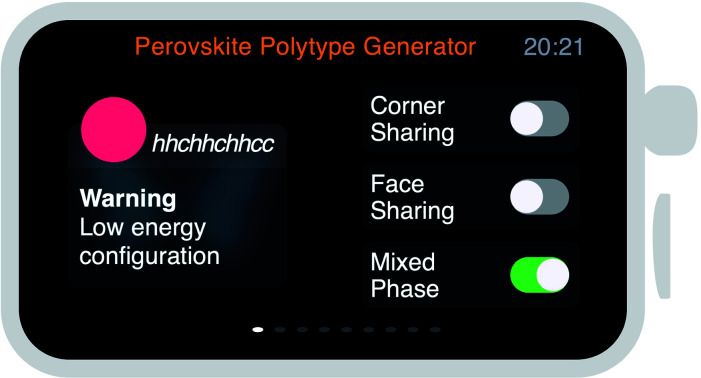

## 1. Introduction

Perovskite is an important and flexible structure type in solid-state chemistry and physics.^1–4^ Metal halide perovskites have attracted significant attention as photovoltaic materials since the first report of a photoactive absorber in 2009,^5^ with the power conversion efficiency (PCE) climbing up from the 3.8% to the current record of 25.5%.^6^ One key for improving solar cell technologies based on the hybrid organic–inorganic perovskites such as MAPbI_3_ (MA = CH_3_NH_3_^+^) and FAPbI_3_ (FA = CH(NH_2_)_2_^+^) or the inorganic lead halide perovskites such as CsPbBr_3_ and CsPbI_3_, is to synthesize large-area single-crystal perovskite films, which is challenging due to intense phase competition.

MAPbI_3_ undergoes degradation to release CH_3_I and NH_3_ gas at temperatures as low as 80 °C,^7^ posing a challenge to long-term stability. The compositions used in the state-of-the-art perovskite solar cells are shifting to incorporate FA^+^ and Cs^+^ as A-site cations, with reported PCEs up to 25.2%^8^ and 20.4%,^9^ respectively. They are comparatively more stable than MAPbI_3_ at elevated temperatures. However, the photoactive black phases (cubic α-phase) of both FAPbI_3_ and CsPbI_3_ can spontaneously transform into photoinactive, non-perovskite yellow phases (hexagonal δ-phase FAPbI_3_; orthorhombic CsPbI_3_) at room temperature.^10–12^ These transformations involve the formation of multiple intermediate phases.^13,14^

Phases can coexist in as-synthesized perovskite crystals, for instance, the presence of cubic and hexagonal domains in a FAPbI_3_ nanowire was confirmed with *in situ* optical micro-spectroscopy.^15^ The authors proposed a disordered amorphous interfacial layer at the phase boundary where the octahedra are distorted and tilted. Recently the mixed perovskite MA_1−*x*_FA_*x*_PbI_3_ (*x* = 0.5, 1) was also reported with characterisation evidencing the appearance of an intergrain 2H phase.^16^ On the other hand, the coexistence of cubic and hexagonal phases is often observed for oxide perovskites. For instance, various mixed (ordered or disordered interleaved) packing of hexagonal and cubic layers in BaNiO_3_, BaCrO_3_, BaMnO_3_, and BaRuO_3_ can form due to the small energetic difference between arrangements, giving rise to strong polytypism.^17,18^ Polytype engineering can enable novel electronic and ion transport properties as demonstrated for hexagonal oxide perovskites in a range of energy-related technologies.^19^

Similarly, polytypism of lead halide perovskites has been shown to be energetically accessible. First-principles calculations revealed that the formation enthalpy of δ-FAPbI_3_ was lower by about 70 meV per formula unit than α-FAPbI_3_ at 0 K.^20^ Our density functional theory (DFT) calculations, reported below, also confirmed that the formation enthalpy of a hexagonal CsPbBr_3_/CsPbI_3_ phase is 40 meV/90 meV per formula unit higher/lower than the corresponding cubic phases at 0 K.^21^ It has been reported that the black to yellow phase transitions in FAPbI_3_ and CsPbI_3_ are entropy-driven and allow for kinetic trapping of intermediate phases.^22^ Another aspect is the small lattice mismatch along the 〈111〉 stacking direction between the hexagonal and cubic polytypes, which is just 4.7% for CsPbI_3_ and 5.4% for CsPbBr_3_, according to our calculations.

Due to the range of accessible ordered stacking sequences with distinct crystal symmetry and the appearance of stacking faults that break symmetry in halide perovskites, polytypism can bring forth novel physical and chemical phenomena. This includes carrier-blocking effects of hexagonal regions in cubic crystals and a likely contribution to photogenerated carrier recombination in other cases.^21^ In this work, we aim to shed light on the phase space for polytype formation in lead halide perovskites. To overcome the limitations of standard modelling approaches, we build a workflow based on an evolutionary exploration of a model crystal Hamiltonian. We provide insight into the ordering trends and ground-state structures of CsPbX_3_ as a prototype system. Universal features such as the accessible layer arrangements and symmetry forbidden sequences are reported.

## 2. Computational methods

We used a combination of computer simulation approaches in this study. DFT can provide reliable first-principles total energies, but is limited to relatively small polytype stacking sequences. For this reason, a model Hamiltonian approach was employed to rapidly predict polytype energies and assess the extent of polytype disorder. Afterwards, a genetic algorithm with optimisation functions derived from the model Hamiltonian was built to rapidly converge the searching of low energy configurations.

### Training data

For each polytype in the training set, DFT calculations were performed using the Vienna *Ab initio* Simulation Package (VASP)^23,24^ with the standard frozen-core projector augmented-wave (PAW)^25,26^ method. The cutoff energy for basis functions was 420 eV. The generalized gradient approximation (GGA)^27^ of the Perdew–Burke–Ernzerhof functional for solids (PBEsol)^28^ was used for the exchange-correlation. We used a dense 7 × 7 × 5 *k*-point mesh for structural relaxation until all atoms were relaxed with Hellmann–Feynman forces below 0.01 eV Å^−1^.

### Model construction

#### Ising-type model Hamiltonian

To get the interaction coefficients for the model Hamiltonian, the summation of each correlation function, ∑_*i*_*σ*_*i*_, ∑_*i*_*σ*_*i*_*σ*_*i*+1_, …, was numerically calculated. Afterwards, the corresponding interaction coefficients, *J*_0_, *J*_1_, …, were generated by solving the linear matrix to get the least-squares solutions.

### Genetic algorithm

To quickly identify the ground-state stacking sequences for a given layer number, a genetic algorithm was designed by setting the model Hamiltonian as the objective function. The parameters for running this algorithm include the maximum number of iteration, population size, mutation probability, elite ratio, crossover probability, and parental portion. These parameters can be tuned to monitor and optimise the performance of the algorithm. The converged values are given in the results section.

## 3. Results

### Thermodynamic stability of CsPbX_3_ polytypes

The thermodynamic stability of a CsPbX_3_ polytype can be evaluated by its ordering energy. Along the stacking chain of a polytype, two adjacent PbX_6_-octahedra are connected either with a face-sharing (*h*) or a corner-sharing (*c*) configuration, forming a unique stacking sequence for each polytype, which in turn determines the interaction between layers and its formability. Given a random CsPbX_3_ polytype with *N* layers, its ordering energy (*E*_O_) is defined as1
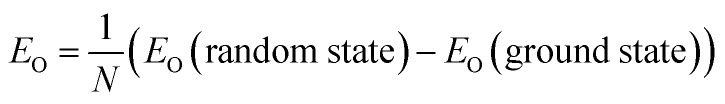


The ordering energy is determined by a combination of electrostatics and crystal strain. Low ordering energy usually indicates the ease of formation. Though similar, the ground state structures of CsPbBr_3_ and CsPbI_3_ polytypes are starkly different, allowing us to probe the chemistry of ordering ions.

For an ionic crystal, the Madelung energy based on point charges should fully describe phase energetics. However, we found that CsPbX_3_ is beyond this regime, due to the contribution from the polarisation of ions. As shown in Fig. 2, under the perfect ionic lattice assumption, the Madelung energies of all the polytypes for the two halide perovskites CsPbBr_3_ and CsPbI_3_ were calculated, we found that the most stable phase should be cubic 3C. Moreover, the Madelung ordering energies *E*_O,Madelung_ for the two sets of halide perovskite polytypes are lowered dramatically, by 2.51 eV/layer (CsPbBr_3_) and 2.55 eV/layer (CsPbI_3_) respectively, from the hexagonal 2H phase to the cubic 3C phase, indicating that the electrostatic Coulomb energies do influence the ordering preferences. ^29^ However, as also shown in Fig. 2, first-principles calculations revealed that the most energetically stable phase for CsPbI_3_ is 2H, while it is 3C for CsPbBr_3_. The ordering energies *E*_O,DFT_ are less than 100 meV/layer for both halides, with *E*_O,DFT_(CsPbI_3_) = 90 meV and *E*_O,DFT_(CsPbBr_3_) = 40 meV between the 2H and 3C phase. The greatly reduced ordering energies mean that the bare coulombic interactions are regulated and screened due to the polarisation of ions. Moreover, the reversed order of stability in CsPbI_3_ and CsPbBr_3_ polytypes indicates the non-negligible role of anion polarisation; although we note that Pb^2+^ is also highly polarisable due to its lone pair (6s^2^) electrons, which contributes to the ionic dielectric response.^30^

**Fig. 1:**
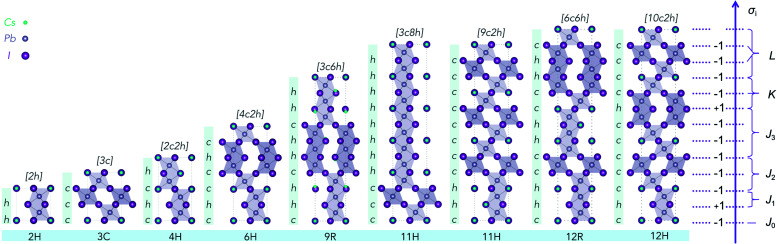
Illustration of nine CsPbI_3_ polytypes with low periodicity (≤12 layers). The cubic perovskite (3C) consists of pure corner-sharing PbX_6_ octahedra; and the hexagonal (2H) structure consists of pure face-sharing octahedral network. Other polytypes in between 2H and 3C are shown and distinguished according to the halide in each layer being corner-sharing (*c*) or face-sharing (*h*). Note that the stacking axis corresponds to the 〈111〉 direction of the cubic unit cell. The labels on the right side correspond to the pseudo-spin (+1 for *h*, −1 for *c*) in the Ising-type model description of the 12H structure. Different interlayer correlations coefficients are labeled with *J*, *K* and *L*, according to their specific interaction types.

**Fig. 2:**
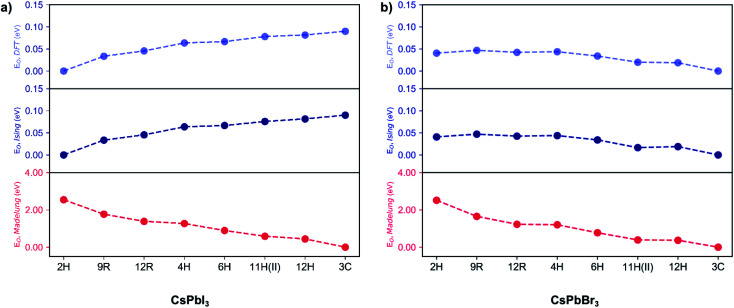
Ordering energies per layer (*E*_O_) for the CsPbX_3_ polytypes. (a) Ordering energies *E*_O,DFT_ computed by DFT (light blue), *E*_O,Ising_ fitted with an Ising-type model Hamiltonian (blue) and *E*_O,Madelung_ evaluated by the Madelung energies (red) for all the fully relaxed CsPbI_3_ polytypes. Reference ground state: the 2H phase. (b) Ordering energies *E*_O,DFT_ computed by DFT (light blue), *E*_O,Ising_ fitted with an Ising-type model Hamiltonian (blue) and *E*_O,Madelung_ evaluated by the Madelung energies (red) for all the fully relaxed CsPbBr_3_ polytypes. Reference ground state: the 3C phase.

### Ising-type model Hamiltonian

While first-principles calculations can provide reliable total energies, they are limited to relatively small stacking sequences and can only provide a fragmentary picture of the physics of ordering. The other hurdle further stops us from using DFT calculations to explore the phase space directly is the explosion of combinations. Structurally, the ordering of *h* and *c* in the stacking chain of a polytype shows a binary nature for each layer. If a polytype has *N* layers, the possible stacking sequences reach 2^*N*^.

To overcome this problem, an Ising-type Hamiltonian was built to calculate polytype energies. This model Hamiltonian can not only be used to compute the random-state energies for the 2^*N*^ stacking sequences of *h* and *c*, but also implicitly includes a full set of chemical interactions between the neighbouring layers, manifested by a series of interaction coefficients and their corresponding correlation functions. In Fig. 1, we showed the mapping of a stacking sequence (12H) onto an Ising-type model with *σ*_*i*_ = 1 for a face-sharing octahedra pair and *σ*_*i*_ = −1 for a corner-sharing octahedra pair. The first, second and third nearest neighbour interactions are included during the model construction. Thereby, for CsPbX_3_ polytypes, the random-state total energy of a polytype with *N* layers can be written as2
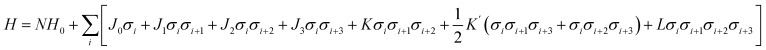
where *N* denotes the number of layers, *H*_0_ is the total energy per layer in the absence of layer interactions, *i* is an index of the layers from 1 to *N*, and *J*, *K*, *L* are interaction coefficients, which can be further obtained by fitting the Hamiltonian to the DFT calculated total energies.

In 1988, Plumer generated an approximate phase diagram for possible ground-state structures of ABX_3_ perovskite polytypes.^31^ In this model, limited by available data at the time, the ground-state structures for both CsPbI_3_ and CsPbBr_3_ should be 3C, concluded by the sign of their coefficients. However, first-principles calculations have confirmed that, even though both CsPbI_3_ and CsPbBr_3_ belong to the ABX_3_ type perovskite and have similar interaction coefficients, the most stable phase for CsPbI_3_ is 2H, while for CsPbBr_3_ it is 3C. This conclusion reflects the underlying chemical complexity and how the choice of elements can play an important role to alter the stability of polytypes with similar stacking sequences.

To obtain the interaction coefficients for CsPbX_3_, total energies of nine polytype phases (2H (2*h*), 3C (3*c*), 4H (2*c*2*h*), 6H (4*c*2*h*), 9R (3*c*6*h*), 11H (3*c*8*h*), 11H (9*c*2*h*), 12R (6*c*6*h*), 12H (10*c*2*h*)) with low periodicity (*N* ≤ 12) for both CsPbI_3_ and CsPbBr_3_ were calculated. During the coefficients fitting stage, we randomly chose eight out of the nine structures for parameter fitting in accordance with the eight unknowns (*H*_0_ and seven coefficients) in the model Hamiltonian, by solving the linear equations. The resulting interaction coefficients for CsPbI_3_ and CsPbBr_3_ are listed in Table 1.

**Table 1:** Interaction coefficients in the Ising-type Hamiltonian for CsPbI_3_ and CsPbBr_3_ polytypes (unit: eV)

CsPbX_3_	*H*_0_	*J*_0_	*J*_1_	*J*_2_	*J*_3_	*K*	*K*′	*L*
CsPbI_3_	−11.243	2.015	1.024	0.004	−1.034	0.0011	−2.061	−1.033
CsPbBr_3_	−12.865	2.353	1.157	−0.005	−1.169	0.0002	−2.333	−1.167

Following this procedure, the model Hamiltonian calculated stability trends, denoted as the ordering energies *E*_O,Ising_ for both CsPbBr_3_ and CsPbI_3_ in Fig. 2, in agreement with the DFT results *E*_O,DFT_. The Ising-type model Hamiltonian is found to work well, with the largest fitting error being negligible at just 3 meV/layer for the 11H(II, 9*c*2*h*)-CsPbBr_3_ polytype and 2 meV/layer for the 11H(II, 9*c*2*h*)-CsPbI_3_ polytype. Furthermore, the model Hamiltonian predictions have been checked with new (unseen) polytype data, which also confirms the high prediction accuracy of the model Hamiltonian (Table S1†).

The impact of ion choice and ordering for tuning the stability of different stacking sequences is contained within the eight interaction coefficients and their corresponding correlation functions. For instance, the *H*_0_ value for the CsPbBr_3_ stacking sequences is about 1.62 eV/layer lower in energy than that for the CsPbI_3_ polytypes. Physically, the *H*_0_ can be viewed as the energy of the suspended monolayer of octahedra. The more negative *H*_0_ value in CsPbBr_3_ polytypes shows that Br^−^ stabilises the perovskite structure more than I^−^, in consensus with the conclusion drawn from the perspective of tolerance factors that cubic CsPbBr_3_ has a more ideal Goldschmidt tolerance factor (*t*(CsPbBr_3_) = 0.92; *t*(CsPbI_3_) = 0.89). This highlights the key anion role in determining polytype stability. The additional chemical contributions are quantified by the seven interlayer correlation-related coefficients in the model Hamiltonian, which act as gears to tune the stability of a given polytype.

The most striking parameters are the small values for *J*_2_ and *K*, being only several meV. As shown in Fig. 1, *J*_2_ denotes the interaction between a layer and its second nearest neighbour, and *K* denotes the interaction between a group of three neighbouring layers. However, it is not the absolute values of the interaction coefficients that dominates the final ordering effect. The interaction coefficients matter only when the stacking sequence allows it. This can be understood from two aspects. One is that the stacking sequence determines the weight of each type of interaction in the model Hamiltonian through the correlation functions: ∑_*i*_*σ*_*i*_, ∑_*i*_*σ*_*i*_*σ*_*i*+1_, …; and the other reason is, the impact of a sole interaction coefficient can only be pronounced when such interaction is not quantitatively counterbalanced by other interactions in a given stacking sequence. For instance, for the CsPbX_3_ polytypes, the total energies of 2H and 3C phases per unit layer are

3 *H*_2H_/2 = *H*_0_ + *J*_0_ + *J*_1_ + *J*_2_ + *J*_3_ + *K* + *K*′ + *L*

4 *H*_3C_/3 = *H*_0_ − *J*_0_ + *J*_1_ + *J*_2_ + *J*_3_ − *K* − *K*′ + *L*

Due to the special stacking sequences for 2H and 3C, the ordering effect of their stacking sequences give an equal weight for different interlayer interactions, which only differ in sign. With the absolute values provided in Table 1, the impact of *J*_2_ and *K* become pronounced because the absolute total contribution of interlayer interactions is at the 10 meV level, making the influence from the *J*_2_ and *K* related terms non-negligible. Moreover, from the model Hamiltonian, why the ground state structures for CsPbBr_3_ and CsPbI_3_ polytypes prefer different stacking sequences can also be explained. By subtracting eqn (3) and (4), the energy difference between the 2H and 3C phases per layer is

5 Δ*E*_2H–3C_ = 2*J*_0_ + 2*K* + 2*K*′

Consequently, the three interlayer interactions corresponding to *J*_0_, *K* and *K*′ are responsible for the ordering energy between the 2H and 3C phases. For magnetic systems, *J*_0_ is viewed as the Zeeman-like term linear in *σ*, representing an externally applied magnetic field pointing along the stacking direction, however in this pseudo-spin model Hamiltonian for polytypism, *J*_0_ is a phenomenological parameter with no implicit physical meaning and if the numbers of *h* and *c* are equal in a stacking sequence, the weight for *J*_0_ will become zero and will not contribute to the total energy. Different from *K* explained previously, *K*′ corresponds to the group interaction of three layers consisting of a pair of nearest neighbouring layers and one layer that is the second nearest neighbour to any of them. As a consequence, the ordering energy between the 2H and 3C phases could be quantitatively decided. Taking the 3C phase as the reference, for CsPbI_3_ Δ*E*_2H–3C_ is −0.09 eV/layer, meaning that the 2H phase is 90 meV/layer more stable than the 3C phase. For CsPbBr_3_, the Δ*E*_2H–3C_ is 0.04 eV/layer, meaning that the 3C phase is 40 meV/layer more stable than its 2H phase. This result arises from the more negative value of *K*′ for CsPbI_3_ polytypes, which alters the ground-state structures. Such more negative value of *K*′ may be also related with the higher polarizability of I^−^ compared to Br^−^.

### Distinct polytypes for an *N* layer polytype *h*_n_*c*_m_

The Ising-type model Hamiltonian opens up a fast means to compute the energy for an arbitrary *h*_n_*c*_m_ polytype. The only computing cost is from the summation over pseudo-spin correlation functions determined by the stacking sequence. An expression to calculate the number of distinct polytypes for an *N* layer polytype *h*_n_*c*_m_, including periodic boundary conditions, takes the form6
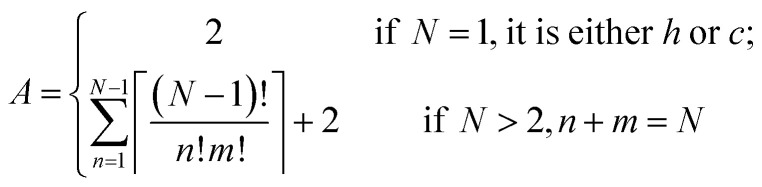
where *A* represents the number of distinct arrangements, *N* is the layer number, *n* and *m* are the number of face-sharing (*h*) and corner-sharing (*c*) layers along the stacking sequence; ⌈⌉ denotes the ceiling of the whole fraction.

Accordingly, we listed the number of distinct arrangement of *h* and *c* for CsPbX_3_ polytypes in Table 2. For instance, a polytype with 2 layers can have 3 distinct arrangements: *hh*, *cc* and *hc* or *ch*; a polytype with 3 layers can have 4 distinct arrangements: *hhh*, *hhc*, *hcc* and *ccc*. With the constraints of periodicity, the space of distinct arrangements of *h* and *c* for CsPbX_3_ polytypes increases slower than the exponential explosion to the 2^*N*^ for a linear chain of pseudo-spin. There are only 60 distinct arrangements for a 9 layer polytype, which should be 2^9^ = 512 without periodic boundary conditions. Similarly, the space of distinct arrangements for a 12 layer polytype also decreases dramatically by 1–311/(2^12^) = 92.4%.

**Table 2:** Distinct arrangements for ABX_3_ perovskite polytypes with low periodicity (≤12 layers)

Layer number (*N*)	2	3	4	6	9	11	12
Arrangements	3	4	6	14	60	186	311
Representative stacking sequence	2H	3C	4H	6H	9R	11H(I),11H(II)	12H,12R

The determination of the distinct polytype set is instructive. Although the energy of a specific polytype *h*_n_*c*_m_ can be computed using the model Hamiltonian swiftly, only with the full set of the distinct polytypes, can we grasp the complete thermodynamic landscape of the polytypism. More generally, the number of distinct arrangements determines the number and degeneracy of energy levels that a polytype *h*_n_*c*_m_ can possess.

### Low energy polytypes

Here, low energy polytypes refer to polytypes whose ordering energies are within the range of ordering energies between the 2H and 3C phases of CsPbI_3_ and CsPbBr_3_. These are 90 meV/layer and 40 meV/layer, respectively. In experiments, during the kinetically controlled growth of CsPbI_3_ and CsPbBr_3_ crystals, usually the low energy structures would dominate. Along the stacking sequence of the cubic CsPbX_3_ lattice, the thickness of an octahedral monolayer is around 3.55 Å for the CsPbBr_3_ and 3.75 Å the for CsPbI_3_. Thus the domain of a 12 layer stacking faults will be about 4.3–4.5 nm in size, both being much smaller than the typical grain sizes of the cubic phase CsPbI_3_ and CsPbBr_3_,^32,33^ which can extend to microns.

A genetic algorithm was adopted to provide an efficient way to locate the ground state structures of polytypes without calculating all energies. At most, it would take 2^12^ = 4096 trials for 12-layer polytypes, but there is an interest to extend to even larger structures in the future. According to evolutionary theory, the gene of a species is a unique DNA sequence. Given the binary nature of each stacking layer, the random stacking sequence of a polytype can also be generated under the biologically inspired operators such as mutation, crossover and selection. Not only that, but also the central idea of natural evolution, ‘survival of the fittest’, is also implemented in genetic algorithms tailored for optimisation problems. The core of a genetic algorithm is the objective function, which here is the Ising-type Hamiltonian for CsPbI_3_ and CsPbBr_3_,7
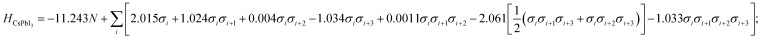
8
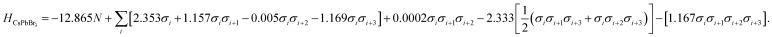


**Fig. 3:**
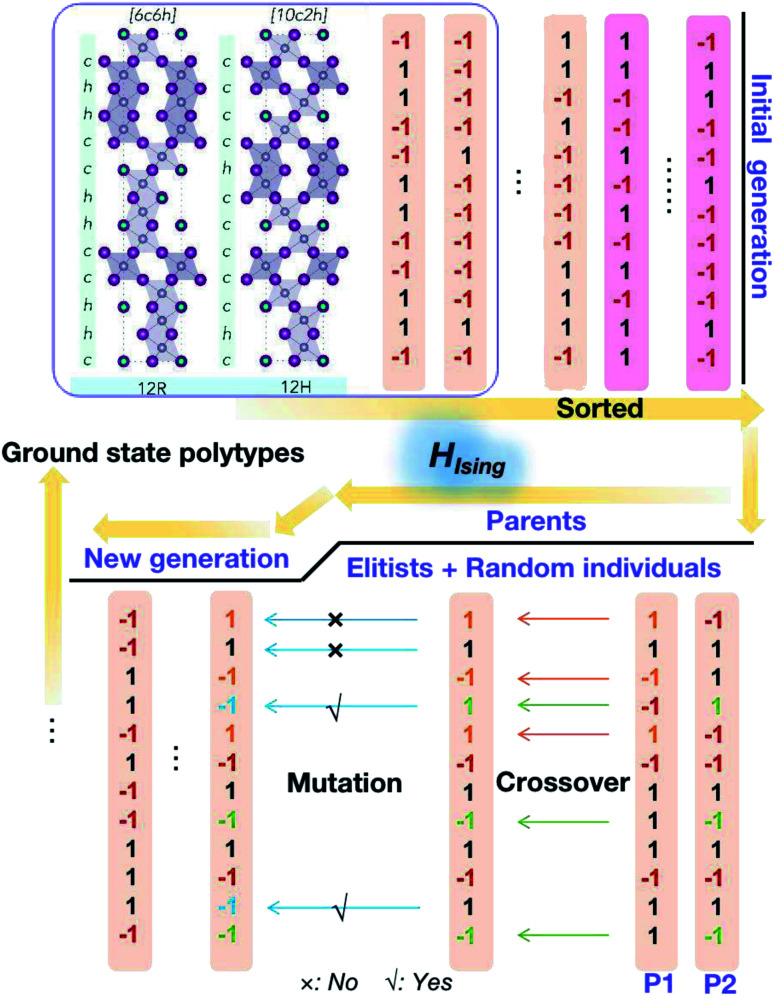
Schematic of the genetic algorithm used to explore the phase space for perovskite polytypes.

During the optimisation process, because of the binary nature of each layer in the stacking chain, the initial population is generated by a random choice between −1 and 1 for each component layer (see Fig. 3). Then the fitness scores, *i.e.* values of the objective function corresponding to different stacking sequences, are sorted and normalized. Afterwards, the cumulative probabilities for the initial population are calculated and used for the parent selection. As a result, the parents are formed of elitists and individuals randomly selected from the current population until the set population size is reached. Then, two basic operators are used to generate the new generation: crossover and mutation. The crossover sites are chosen randomly and the number of them is determined by the crossover probability. Here the crossover happens in a uniform way by ‘flipping a coin’ for each site of the parents to decide whether or not it will be included in the next generation; and the mutation takes place randomly within the given mutation probability. Finally amongst the new generation, the new best pseudo-spin sequence to minimize the objective function is generated and compared with the previous generations, which guarantees the algorithm to keep descending to the global minimum for the objective function. The optimisation process will stop when it reaches the set maximum number of iterations.

**Fig. 4:**
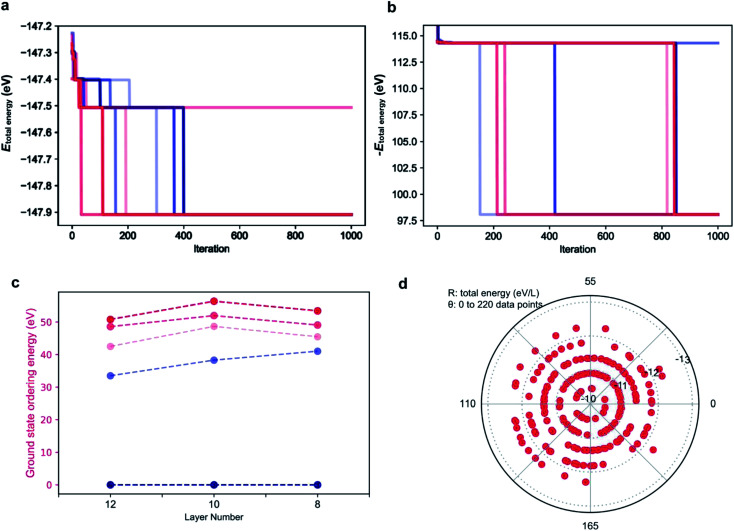
Performance of the genetic algorithm for exploring polytype structures. (a) convergence diagram of the genetic algorithm to find the stacking sequence with the lowest total energy of the 12 layer polytypes. To show the plateaus more clearly, parameters are intentionally chosen to slow down the convergence. Different colors correspond to different rounds of running with the same parameters: max_num_iteration: 1000; population_size: 10; mutation_probability: 0.6; elit_ratio: 0.2; crossover_probability: 0.1; parents_portion: 0.6. (b) convergence diagram of the genetic algorithm to find the stacking sequence with the highest total energy of 12 layer polytypes. Parameters are the same as that in (a). (c) ordering energies for typical ground state polytype structures (grey) for CsPbI_3_ with layer number 12, 10, 8. Reference ground state: the 2H phase. (d) distribution of total energies for 220 random polytypes calculated using the model Hamiltonian. Layer number: 12.

As the parameters used for the genetic algorithm affect the speed and output, a series of tests was conducted to detect the model sensitivity. Taking the 12 layer CsPbI_3_ polytype as an example, the genetic algorithm converges to the most stable phase of the 12 layer CsPbI_3_ in only two seconds with the output stacking sequence {1, 1, 1, 1, 1, 1, 1, 1, 1, 1, 1, 1}, *i.e.* the 2H phase, consist with the DFT results. Interestingly, as shown in Fig. 4a, there are plateaus during the convergence, corresponding to energy levels for distinct polytypes. This also means that other competitive structures can be extracted from the plateaus by tuning the parameters in running the algorithm. Fig. 4c shows the selected ground state structures for the 12, 10, 8 layer CsPbI_3_ polytypes and their ordering energies compared with the hexagonal 2H phase. Detailed stacking sequences for these ground state polytypes are listed in Table 3. For instance, the 12 layer ground state with an ordering energy of 33 meV per layer in Fig. 4c corresponds to the 12R phase. Such low energy polytypes should be accessible thermodynamically. By considering the thermal energy at room temperature, polytypes with ordering energies lower than 25 meV/layer are more likely to be detected in experimental samples. For example, the 18-layer 2*c*7*h*2*c*7*h*-CsPbI_3_ polytype with a 23.5 meV/layer ordering energy is a candidate phase.

**Table 3:** Low energy structures for 12, 10, 8 layer CsPbI_3_ polytypes. Here, 2H refers to a sequence of all *h*, whereas 12R refers to the case of *hhcchhcchhcc*. The corresponding ordering energies can be read from Fig. 4c

Layer number	Candidate structures				
12	2H	12R	*hhchhcchhchh*	*ccchhchhchhc*	*hchhcchchhcc*
10	2H	*hhchhchhcc*	*hhcchhcchc*	*ccchhchchh*	*hhcchhcccc*
8	2H	*hhchhchc*	*hhcchhcc*	*hccchhch*	*chchhchc*

The genetic algorithm can also be used to find the highest energy phases by flipping the sign of the objective function. In principle, the pure 3C phase should be the highest energy. However, as shown in Fig. 4b for the 12 layer polytypes, the output total energy is higher with the global maximum being the stacking sequence of *hhhchhhchhhc*. The origin of such high energy phases is discussed in the next section.

To validate the predictions, 220 stacking sequences from the 12 layer CsPbI_3_ polytypes were randomly sampled and their total energies were calculated. As shown in Fig. 4d, a polar coordinate was used to distribute the 220 total energies, with the radius denoting the total energy per layer and the angle representing each polytype. Similar to the plateaus that emerged during the genetic search, the total energies for these 220 polytypes are also discretely distributed, mainly falling upon five energy levels. Taking the total energy per layer of the pure 3C CsPbI_3_ as the reference, which is −12.23 eV/layer, most of the total energies fall in the inner energy levels, indicating that these stacking sequences will be hard to form. Moreover, typical sequences exhibiting abnormally high total energies per layer are extracted from the learning paleatus, such as *hhhc*, *hhhccc*, *hchhhc*, *hhhhcc* and *hhhhhc*. These sequences can be viewed as the high energy gene for a polytype, which are not favored during the evolution, for instance, *hhhc* and *hhhhchhhhchhhc* were found to be unstable. Such high energy gene sequences led us to identify symmetry forbidden sequences, which further decrease the number of available arrangements for *h* and *c*.

### Symmetry forbidden sequences and the electronic impact of stacking sequences

**Fig. 5:**
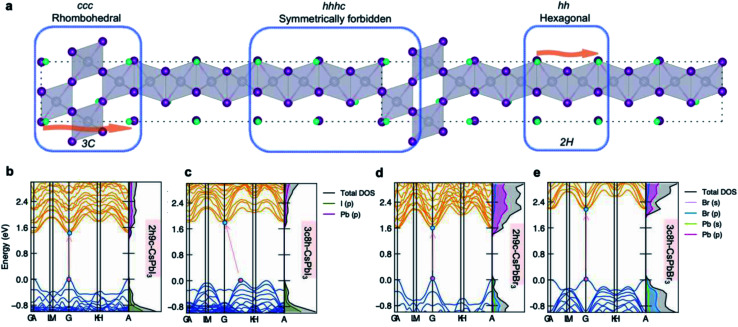
(a) A schematic polytype containing the *ccc* (3C), *hh* (2H) and the symmetry forbidden *hhhc* sequences to show how translational symmetry is preserved/broken. The orange arrow points from start to the end of a stacking sequence with translational symmetry. (b–e) Impact of stacking sequence on the electronic structures of (b) 2*h*9*c*-CsPbI_3_, (c) 3*c*8*h*-CsPbI_3_, (d) 2*h*9*c*-CsPbBr_3_, (e) 3*c*8*h*-CsPbBr_3_ polytypes.

The stacking sequences predicted to be highly unfavourable are in fact forbidden by crystal symmetry. The root cause is the mismatch between different counts of *h* and *c* layers in a stacking sequence. Originally, the representation that we use for the 3C unit cell is rhombohedral (*R*3̄*m*), while the 2H unit cell is hexagonal (*P*6_3_/*mmc*). However, as shown in Fig. 5a, for the sequence *hhhc*, there is no translational symmetry as the start and end of the cell are located at different atomic sites. While the energy of these sequences can be calculated from the model Hamiltonian, which has no atomic resolution, they cannot be mapped onto a valid atomistic supercell. Such sequences should be at least doubled to form a valid centrosymmetric repeat unit. Therefore, for the sequence *hhhc*, its lowest periodicity is *hhhchhhc*.

To preserve translational symmetry for an arbitrary stacking sequence *h*_n_*c*_m_, its minimum periodicity of layer number *P* can be determined by the following three rules:

(1) If *n* is odd, then *P* = 2(*n* + *m*), the lattice is hexagonal;

(2) If *n* is even, then *P* = 3(*n* + *m*), the lattice is rhombohedral;

(3) If *n* is even and *m* is 3*X*, *X* is zero or an positive integer, then *P* = (*n* + *m*), the lattice is rhombohedral. Thus, the genetically unfavorable stacking sequences, *i.e. hhhc*, *hhhccc*, *hchhhc*, *hhhhcc*, *hhhhhc*, are symmetry forbidden. These can only be component sequences for larger lattices and cannot exist alone, so the *hhhc* should be doubled into the centrosymmetric hexagonal *hhhchhhc* sequence; the *hhhccc* should be doubled into the centrosymmetric hexagonal *hhhccchhhccc*; and the *hchhhc* should be tripled into the rhombohedral *hchhhchchhhchchhhc* sequence and so on. The requirement of translational symmetry further decreases the number of distinct polytypes for an *N* layer polytype *h*_n_*c*_m_.

At last, an example of the impact of stacking sequences on the underlying electronic structures of polytypes is presented in Fig. 5b–e. Taking the 11-layer polytypes as an example, the DFT electronic structures of 2*h*9*c* and 3*c*8*h* polytypes are shown. The effect of stacking sequence manifests itself by altering the band structures for two CsPbI_3_ polytypes, that is, from a direct band gap semiconductor (2*h*9*c*-CsPbI_3_) to an indirect band gap semiconductor (3*c*8*h*-CsPbI_3_). Even though a direct band gap is retained for CsPbBr_3_ polytypes, the 3*c*8*h*-CsPbBr_3_ polytype exhibits a noticeably larger band gap than 2*h*9*c*-CsPbBr_3_. The influence of stacking sequences shows the potential for electronic structures engineering in perovskite polytypes with identical compositions.

## 4. Conclusions

A family of perovskite polytypes can be represented by sequences of the face- and corner-sharing PbX_6_ octahedra. The combinatorial explosion arising from the increasing periodicity of the stacking sequence makes it resource and time demanding to perform exhaustive first-principles calculations. To address this problem, an Ising-type model Hamiltonian was constructed and the total energy for any given stacking sequence can be rapidly computed. A genetic algorithm was then built to find the ground state structures for an *N* layer polytype *h*_n_*c*_m_. The algorithm can converge to the lowest energy sequence in seconds without enumeration over the sequences. Plateaus in the convergence behaviour led us observe symmetry forbidden sequences, which shrinks the possible valid arrangements of *h* and *c* to form polytype structures.

The energetic and configurational features of the polytypism in CsPbX_3_ discussed in this work are also instructive for organic–inorganic systems, an extension which would require additional parameterisation for molecular disorder, for example through an on-site electrostatic model. A strong molecular disorder contribution is expected when the A-site cation is methylammonium (MA^+^) cation, due to its polar nature and relatively fast rotations. The formamidinium (FA^+^) is less polar and with slower rotations so FAPbI polytypes may be expected to behave more similar to CsPbBr_3_, considering both its close to ideal tolerance factor (0.99) and ground state 3C phase.

Finally, we note the polytypes discussed here may appear as stacking faults or inclusions rather than as distinct phases for many halide perovskite compositions. We hope that this work will help to bridge the divide between the real-world complexity and atomistic modelling of halide perovskite crystals, and can support future polytype engineering.

## Data availability

Structure models and the codes used to generate all structures to parameterize the Ising model Hamiltonian are available in an on-line repository at http://doi.org/10.5281/zenodo.5186354.

## Author contributions

Z. L. performed all calculations for model building and testing. J. P. wrote the code for polytype generation. A. W. supervised the project. All authors contributed to the analysis and writing.

## Conflicts of interest

There are no conflicts of interest to declare.

## Supplementary Material

SC-012-D1SC03098A-s001
