# Supplementary material for: Evolutionary exploration of polytypism in lead halide perovskites
Source: Chem Sci. 2021 Aug 10;12(36):12165–73. doi: 10.1039/d1sc03098a (PMC8457387; doi:10.1039/d1sc03098a)
Supplement: SC-012-D1SC03098A-s001 [file SC-012-D1SC03098A-s001.pdf]

Electronic Supplementary Material (ESI) for Chemical Science.
This journal is © The Royal Society of Chemistry 2021

Supporting Information: Evolutionary exploration of polytypism in lead halide perovskites

Zhenzhu Li,† Ji-Sang Park,‡ and Aron Walsh*,†,¶

†*Department of Materials, Imperial College London, London SW7 2AZ, UK*

‡ *Department of Physics, Kyungpook National University, Daegu 41566, Korea*

¶*Department of Materials Science and Engineering, Yonsei University, Seoul 03722, Korea*

E-mail: a.walsh@imperial.ac.uk

Table S1: Comparison of ordering energies for unseen polytypes calculated from DFT and using the pre-trained model Hamiltonian (total energy in eV; ordering energy in meV/layer).

New polytypes	$E_{Total,DFT}$	$E_{Total,Model}$	$E_{Ordering,DFT}$	$E_{Ordering,Model}$
11H(3c8h)-$CsPbI_3$	-135.243	-134.766	30.8	74.2
11H(3c8h)-$CsPbBr_3$	-154.256	-154.371	45.8	35.4
18H(1h8c1h8c)-$CsPbI_3$	-220.376	-220.343	82.6	84.4
